# The Interaction between Seasonality and Pulsed Interventions against Malaria in Their Effects on the Reproduction Number

**DOI:** 10.1371/journal.pcbi.1004057

**Published:** 2015-01-15

**Authors:** Jamie T. Griffin

**Affiliations:** MRC Centre for Outbreak Analysis and Modelling, Department of Infectious Disease Epidemiology, Faculty of Medicine, Imperial College London, London, United Kingdom; Duke University, UNITED STATES

## Abstract

The basic reproduction number (*R*_0_) is an important quantity summarising the dynamics of an infectious disease, as it quantifies how much effort is needed to control transmission. The relative change in *R*_0_ due to an intervention is referred to as the effect size. However malaria and other diseases are often highly seasonal and some interventions have time-varying effects, meaning that simple reproduction number formulae cannot be used. Methods have recently been developed for calculating *R*_0_ for diseases with seasonally varying transmission. I extend those methods to calculate the effect size of repeated rounds of mass drug administration, indoor residual spraying and other interventions against *Plasmodium falciparum* malaria in seasonal settings in Africa. I show that if an intervention reduces transmission from one host to another by a constant factor, then its effect size is the same in a seasonal as in a non-seasonal setting. The optimal time of year for drug administration is in the low season, whereas the best time for indoor residual spraying or a vaccine which reduces infection rates is just before the high season. In general, the impact of time-varying interventions increases with increasing seasonality, if carried out at the optimal time of year. The effect of combinations of interventions that act at different stages of the transmission cycle is roughly the product of the separate effects. However for individual time-varying interventions, it is necessary to use methods such as those developed here rather than inserting the average efficacy into a simple formula.

## Author Summary

Mathematical models of the transmission of malaria and other infectious diseases are helpful for understanding and predicting the impact of interventions. An important summary of the dynamics of these models is the basic reproduction number, defined as the average number of secondary infections resulting from each infection in a susceptible population. The relative change in the reproduction number due to the intervention is known as the effect size. The effect size is often simple to calculate when conditions do not change over time, but not when there is seasonal variation in transmission or when intervention effects vary over time. I show how to numerically calculate the effect size in these cases, and apply the methods described to mass drug administration, indoor residual spraying and other interventions against *Plasmodium falciparum* malaria in Africa. The best time of year for drug administration is in the low season, whereas the best time for indoor residual spraying or a vaccine which reduces infection rates is just before the high season. Once the effect sizes of individual interventions have been calculated, the effect size of a combination can be often be approximated by multiplying the separate effects.

## Introduction

The basic reproduction number (*R*_0_) of an infectious disease is defined as the number of secondary cases produced by typical infected case in an otherwise susceptible population. It is important for disease control as it tells us what magnitude of control effort is needed in order to prevent endemic transmission. Malaria was one of the first diseases in which the importance of *R*_0_ was recognised, and insights based on *R*_0_ informed the campaigns to reduce or eliminate malaria transmission that took place in the 1950 and 60s.

For a given mathematical model of malaria transmission, *R*_0_ can be calculated using formulae of varying complexity, as long as conditions are assumed to be constant over time. However mosquito numbers are highly seasonal in many places, in sub-Saharan Africa primarily due to rainfall. In a seasonal setting there are in general no known explicit formulae for *R*_0_ for vector-borne infections. Recently Bacaër and co-authors have developed approximate formulae for some simple special cases, as well as numerical methods that can be applied more generally [1–3].

The relative change in the reproduction number due to an intervention is called the effect size [4]. This is a particularly useful summary of the impact of an intervention because in a time-constant setting it is often simple to calculate, depending only on the part of the transmission cycle that is affected by the intervention, and under standard assumptions it is independent of the baseline transmission intensity. However, in addition to seasonal variation some interventions are either inherently time-varying, such as mass drug administration (MDA), or in practice have substantially varying efficacy over time, for example the indoor residual spraying of insecticide (IRS).

I show how numerical methods can be applied not only to find *R*_0_ with seasonally varying mosquito numbers, but also to calculate the effect size due to repeated time-varying or pulsed interventions such as IRS or MDA. I then explore the optimal timing of these interventions relative to the peak in mosquito numbers. Finally, I look at how multiple interventions combine in their effect on the reproduction number in a time-varying environment, both for time-constant and time-varying interventions.

## Results

### Effect size

The effect size *E* of an intervention is defined as the ratio of the basic reproduction number *R*_0_ and the reproduction number with the intervention in place, *R_C_*, so that larger values for *E* mean greater efficacy, as in [4].

$$E=\frac{R_{0}}{R_{C}}$$

### Time-constant effects

The reproduction number in a periodic setting can be calculated by classifying infected hosts according to the time of year that they became infected (see the Methods section). This allows us to show that for an effect which does not vary in time and which does not affect the time-course of infectivity of any host (although it may change infectivity by a constant amount), the relative change in the reproduction number is the same as in a time-constant setting.

For example, if there is heterogeneity between people in the rate at which they are bitten by mosquitoes, this increases *R*_0_ by the same amount in a seasonal and non-seasonal environment, by a factor of 1 + *c_v_* ^2^, where *c_v_* is the coefficient of variation of the distribution of relative biting rates. Also, the effect size of an intervention is the same when there is heterogeneity in biting as when there is no heterogeneity, as long as receipt of the intervention is independent of the variation in biting rates.

If the effects of an intervention do not vary over time and it does not affect the time-course of infectivity, then its effect size is the same in a seasonal as in a non-seasonal setting. For example, if there was a vaccine with negligible waning of efficacy, with 100% coverage and which prevents 50% of human infections, then the effect size would be 2.

Interventions such as insecticide-treated nets (ITNs) do affect the time-course of infectivity from mosquitoes to humans, since they shorten the life-span and hence the infectious period of the mosquito, and numerical methods are needed to find *R_C_* and the effect size for ITNs when there is seasonality, or for any intervention whose efficacy varies over time, with or without seasonality.

### Seasonal curves of mosquito numbers

**Figure 1:**
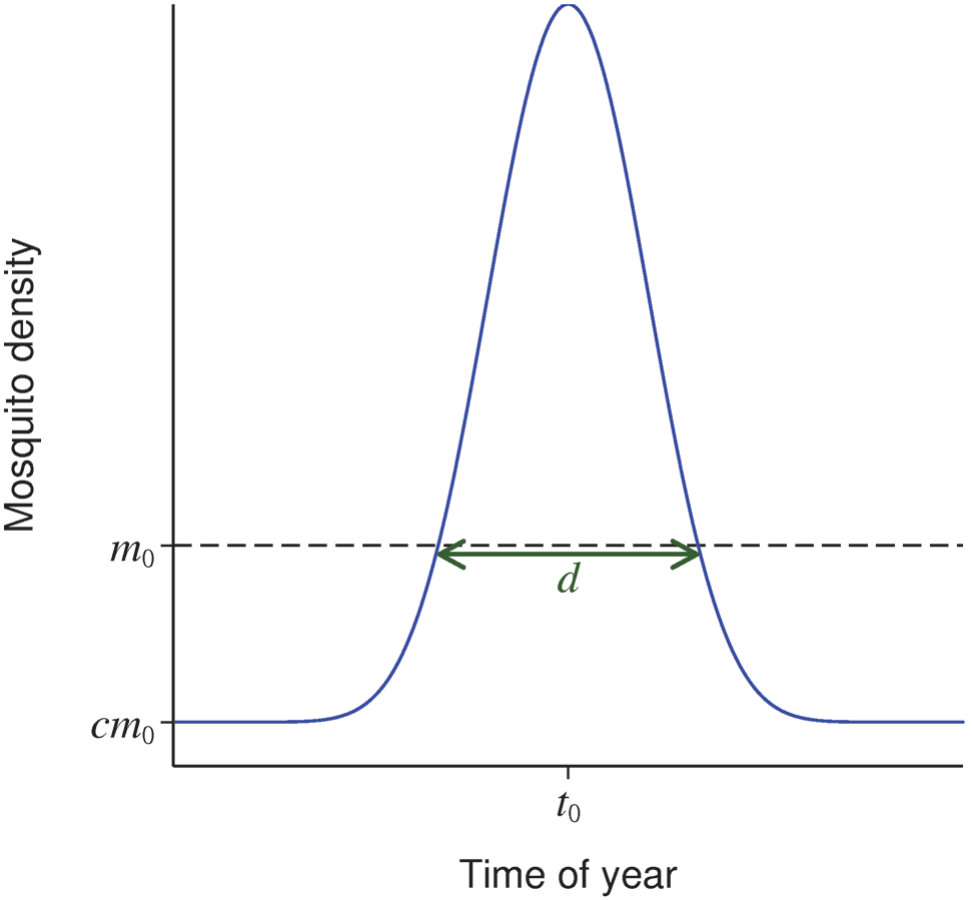
Parametric form for seasonally varying mosquito density. *m*_0_: mean mosquito density; *c*: low season density relative to the mean; *t*_0_: time of peak density; *d*: duration of the high season.

A simple parametric form is used to describe seasonally varying mosquito numbers with a single peak (Fig. 1). *m*_0_ is the mean mosquito density over the year, *c* is the low season density relative to the mean, *t*_0_ is the time of peak density and *d* is the duration of the high season, defined as the period when the density is greater than *m*_0_. The formula is given in equation (6) in the Methods section.

This parametric form was fitted to published datasets of seasonally varying mosquito densities (Methods). Based on these datasets, in the subsequent results to represent a highly seasonal setting the default values used were 3 months for *d* and 0.05 for *c*, with other values also explored.

### Seasonality and R_0_

Bacaër derives in [1] an approximate formula for *R*_0_ when there is sinusoidal seasonal variation, and if the human and mosquito infectious periods are exponentially distributed with no latent periods. In the notation used here, sinusoidal seasonal variation corresponds to a six month high season, and in that case ε in equations 2 and 3 of [1] is equal to 1 – *c* here. The ratio of *R*_0_ with seasonality compared to *R*_0_ in a non-seasonal setting with the same mean mosquito density per person is approximately

$$1-\frac{{\left(1-c\right)}^{2}}{2}\frac{r\mu }{\left({\left(2\pi \right)}^{2}+{\left(r+\mu \right)}^{2}\right)}$$ (1)

where *r* is the human recovery rate and μ is the mosquito death rate, with time units of years. With a delay from mosquito infection to becoming infectious of length τ, then equations 18 and 31 in [1] can be used to show that the relative reduction in *R*_0_ is approximately

$$1+\frac{{\left(1-c\right)}^{2}}{2}ℜ\left(1/z\right)$$ (2)

where *z* = *e^2πiτ^* (1 + *2πi/μ*)(1 + *2πi/r*) – 1, ℜ denotes the real part of a complex number and $i=\sqrt{-1}$.

Unless stated otherwise, in this paper the time-course of human infectiousness to mosquitoes is based on data from malaria-therapy patients as described in the Methods. In this model, the mean human to mosquito generation time is around 70 days.

**Figure 2:**
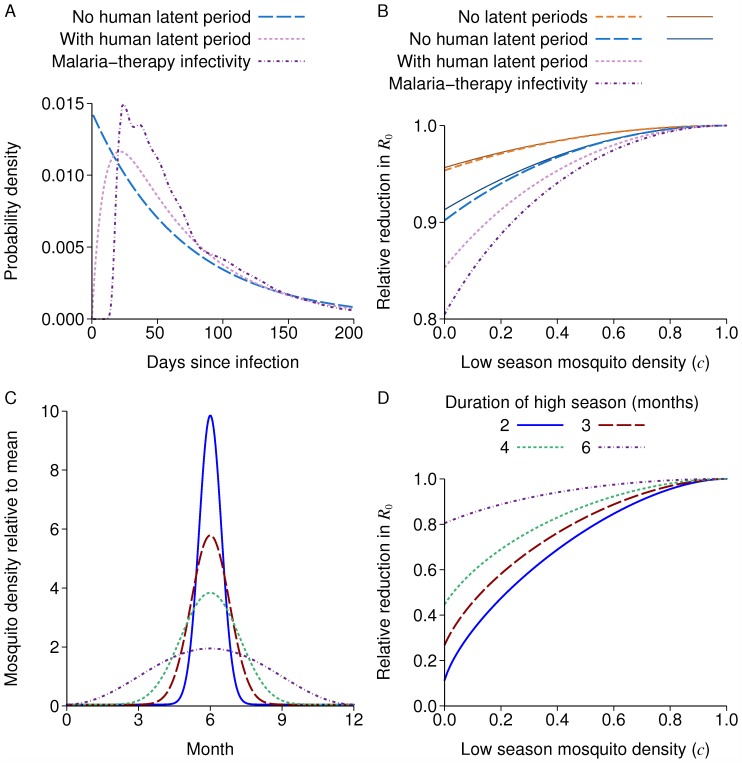
Reduction in R_0_ due to seasonality. A: Human to mosquito generation time distribution for three models. B: *R*_0_ relative to a non-seasonal model with the same mean mosquito density for different transmission models, with sinusoidal seasonal variation. The last three lines all have a mosquito latent period, and correspond to the generation times in A. The thin solid lines are the approximate formulae in equations (1) and (2). C: Seasonal mosquito density with varying duration of high season, and *c* = 0.05. D: *R*_0_ relative to a non-seasonal model with the same mean mosquito density. Colours for the duration of the high season are the same in C and D.

Fig. 2A shows the probability distribution for the human to mosquito infection generation time for this model and two simpler models, an exponential infectious period with mean 70 days, or a latent period with mean 10 days then an infectious period with mean 60 days, both exponential.

Fig. 2B shows the reduction in *R*_0_ with sinusoidal seasonal variation compared to a non-seasonal setting for these three models, each with a mosquito latent period of 10 days fixed duration, plus a model with no latent periods. The formulae of equations (1) and (2) closely approximate the models without and with a mosquito latent period respectively, and no human latent period, even for values of *c* near 0. With human or mosquito latent periods, there is a larger reduction due to seasonality, with a reduction up to 20% for the model used in the rest of this paper.

The reductions when the seasonal curve has a long low season with little transmission are much greater than with sinusoidal seasonal variation (Fig. 2D), with a reduction of up to 70% when there is a three month high season. The effect of increasing seasonality in reducing *R*_0_ can be understood in terms of onward infectivity being wasted from the point of view of the parasite if a host is infected during the high season, but then much of their infectious period is in the low season. Also, if there is no latent period then a host infected near the peak of the high season has some onward infectivity near the peak season too, so less of their onward infectiousness is wasted than if there is a latent period.

### Effect size of time-varying interventions

Time-varying interventions are assumed to be repeated indefinitely in a periodic pattern, with the same cycle of interventions repeated at the same times each year, or in a repeating cycle whose length is a whole number of years. The reproduction numbers without and with the intervention, *R*_0_ and *R_C_* are each found numerically using the method based on the next generation matrix described in the Methods section.

### IRS in a non-seasonal setting

**Figure 3:**
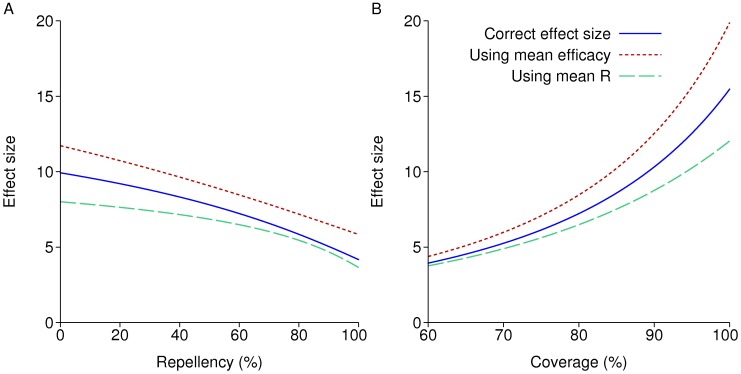
Effect size of repeated annual rounds of IRS in a non-seasonal setting. The solid blue line is the true effect size calculated numerically. The line labelled “Using mean efficacy” is found by plugging the mean efficacy over the year into a reproduction number formula; and the line labelled “Using mean R” is found by plugging the efficacy at each time into a reproduction number formula, and then finding the mean over the year. A: Varying the repellency of the insecticide: lower repellency means greater lethality. B: Varying the coverage.

Fig. 3 shows the effect size of a repeated annual round of IRS in a non-seasonal setting, either keeping coverage at 80% and varying the lethality/repellency balance of the insecticide used, or varying the coverage. Efficacy at both repelling and killing mosquitoes is assumed to decay exponentially with a six month half-life, so that the efficacy relative to the initial value is *e* = 1 immediately after spraying and after one year *e* = 0.25. The blue line is the correct effect size for these model assumptions, with the reproduction number under control *R_C_* calculated numerically using the methods detailed in the Methods section. The more repellent the insecticide the lower the effect size, as mosquitoes are prevented from coming into lethal contact with the insecticide, as previous modelling work has shown [5]. In Fig. 3B and in later results, the repellency is taken to be 60%, unless stated, meaning that before any decay in efficacy, 60% of mosquitoes are repelled without feeding; the remainder feed, and then die if they rest indoors. At later times, the probabilities of being repelled and of dying if not repelled are multiplied by *e*.

The other two lines use a closed form formula for *R*(*e*) at a given efficacy in two different ways. *R*(*e*) is found by calculating the mosquito birth rate, death rate, biting rate on humans in sprayed and unsprayed houses, and equilibrium mosquito density per person if IRS of efficacy *e* is in place, and then plugging these into a reproduction number formula. This is only the true *R_C_* (in a non-seasonal setting) if *e* does not change over time. The line labelled “Using mean efficacy” takes $R_{0}/R(\bar{e})$ as the effect size, where *ē* is the mean efficacy over the year. The line labelled “Using mean R” takes $R_{0}/\bar{R}$ as the effect size, where $\bar{R}$ is the mean over the year of *R*(*e*). These quantities differ from each other because *R*(*e*) is a non-linear function of *e*, and both are substantially different from the true effect size. In particular the true *R_C_* is lower than $\bar{R}$, and so using $\bar{R}$ under-estimates the effect size.

### IRS in a seasonal setting

**Figure 4:**
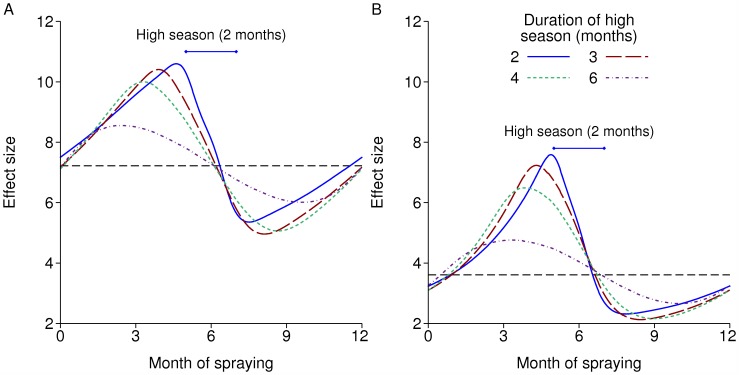
Effect size of IRS with seasonality according to the time of year of spraying. IRS is repeated annually with 80% coverage. Each line is for a different length of high season, all centred around 6 months. The black dashed lines are the effect sizes without seasonality. A: Insecticide with 60% repellency and a 6 month half-life. B: Insecticide with 20% repellency and a 3 month half-life.

There is a marked interaction with seasonality in the effect size of IRS (Fig. 4). Fig. 4A represents an insecticide such as DDT, which repels many mosquitoes, while Fig. 4B represents lambdacyhalothrin, which is less repellent and hence more lethal but has shorter-lived efficacy. If there is a single round each year, the optimal time to spray is just before the high season. In the more seasonal settings, the effect size of just over 10 at this optimal time with DDT is around twice its value at the end of the high season, while with the shorter-lived insecticide, the relative difference between the best and worst times to spray is a factor of more than three.

### MDA

**Figure 5:**
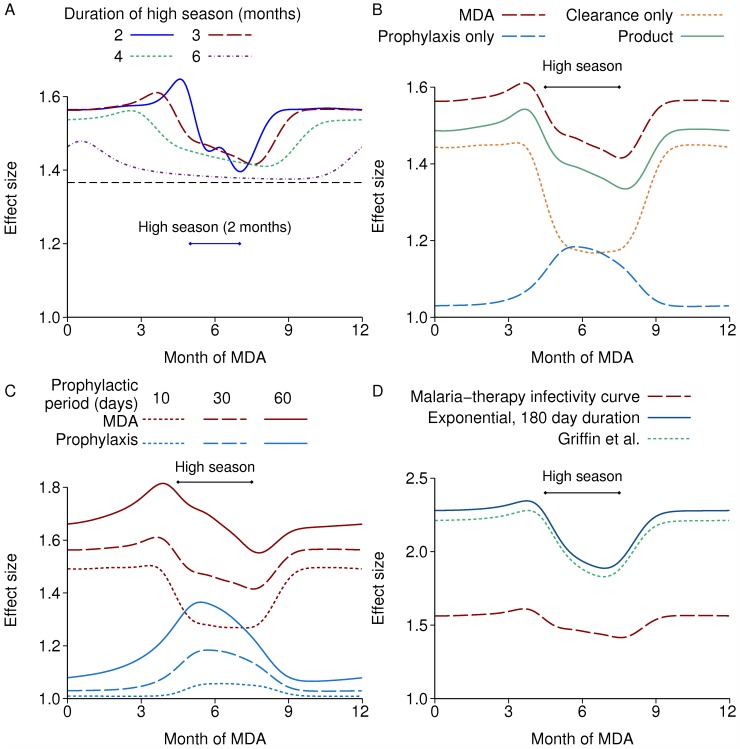
Effect size of repeated annual rounds of MDA at varying times of year. In all cases, the high season is centred at 6 months and MDA is repeated annually with 80% coverage. In B, C and D, the high season is three months long. A: With different lengths of the high season. The black dashed line is the effect size in a non-seasonal setting. B: Components of the effect size. C: Prophylactic and overall MDA effect size with alternative durations of prophylaxis. D: MDA effect size with alternative durations of human infectiousness.

Unlike IRS, MDA is an inherently pulsed intervention, and so there is no equivalent to the mean reproduction number, or the reproduction number using the mean efficacy. Again, I looked at the effect size of a single round each year with 80% coverage. The effect size is much smaller than IRS, below 2. Any time in the low season is better than during the high season, with the period just before the high season being a little better than the rest of the low season (Fig. 5A). If carried out at the best time of year, the effect size increases with shorter high seasons.

The effect can be decomposed into the contributions of clearance of existing infections and prophylaxis against new infections, by redoing the calculation with each effect in turn. Clearance of infections has the larger effect size and is more effective in the low season (Fig. 5B). Infection persists mainly in the human population during the low season, whereas it is also in the mosquito population during the high season, so a larger fraction of the total reservoir of infection is cleared by MDA in the low season. In contrast, prophylaxis is more effective during the high season when most new infections occur. Note that clearance of infections is equivalent in its effect size to mass screening and treatment (MSAT) if all infections are detectable and there are no false positives. This is because for calculation of the reproduction number we only consider a single infectious challenge per person, and so the prophylactic effect of MSAT does not affect *R_C_*, although it does affect outcomes with endemic transmission such as prevalence of infection. The product of the two components’ effect sizes, labelled “Product” in Fig. 5B, is similar to the actual effect size of MDA, but the latter is around 4% higher when there is a three month high season, indicating a slight synergy between the two effects. The mean duration of prophylaxis is assumed to be 30 days, which is at the high end for anti-malarial drugs. Fig. 5C shows the prophylactic and overall effect with 10, 30 or 60 days’ prophylaxis. The maximum prophylactic effect size is comparable to the clearance effect if there is 60 days’ prophylaxis, but the optimal time to treat remains just before the high season.

In the preceding results, human infectiousness to mosquitoes is based on malaria-therapy data, with a mean human to mosquito generation time of around 70 days. If instead, we assume that there is a 10 day latent period in humans followed by an exponentially-distributed infectious period of 180 days’ mean duration, with constant infectiousness during this time, the effect size is much greater, with a maximum effect size of almost 2.3 (Fig. 5D). This is also the case with the model of Griffin et al. using the fitted parameters from [6], which assumes a long period of asymptomatic infection with constant infectiousness. This larger effect size is to be expected, since the reduction in transmission resulting from clearing infections is greater for longer infectious periods. The effect size of IRS was not greatly affected by the duration of the human infectious period (not shown).

### Two rounds of MDA or IRS per year

**Figure 6:**
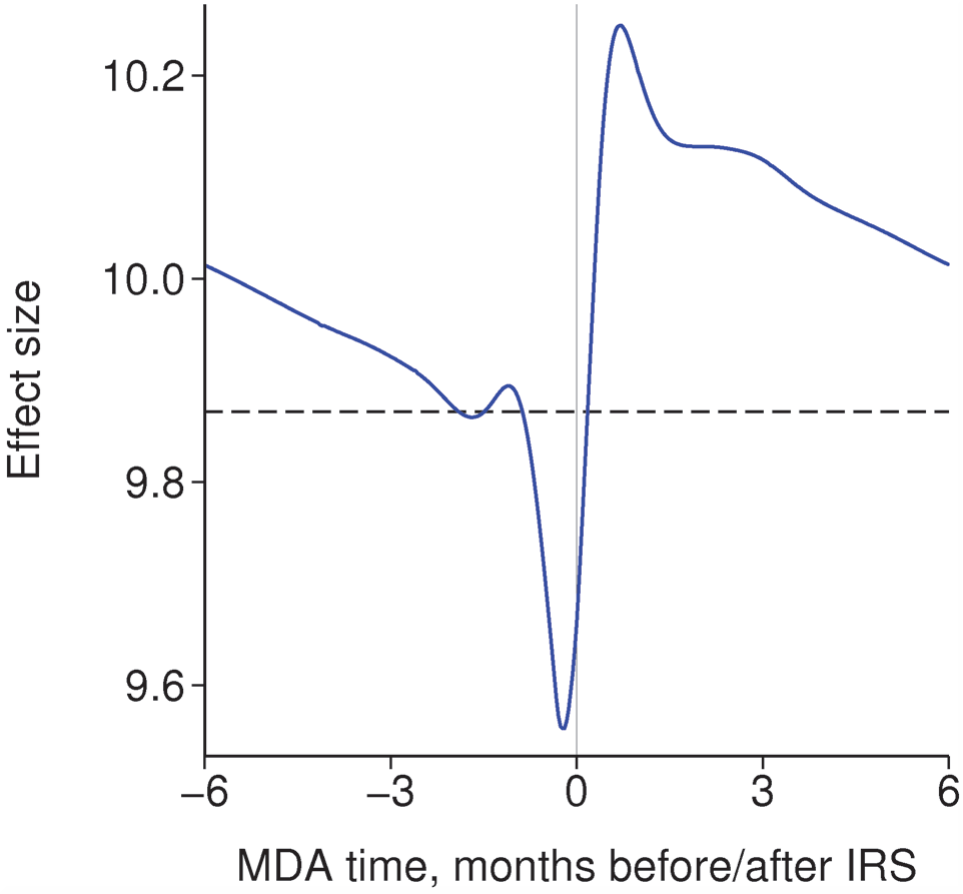
Effect size of annual rounds of both MDA and IRS in a non-seasonal setting according to their timing relative to each other. Each intervention is repeated annually with 80% coverage. The dashed line is the product of the separate effect sizes.

If there is a single round each of IRS and MDA per year, in a non-seasonal setting it is better for MDA to follow just after the IRS round (Fig. 6), which is when transmission has been most reduced by IRS. This is consistent with the effect size of MDA being larger in the low season than in the high season. However, the relative timing only makes a small difference, and the combined effect size is usually within three percent of the product of the effect sizes of each intervention.

**Figure 7:**
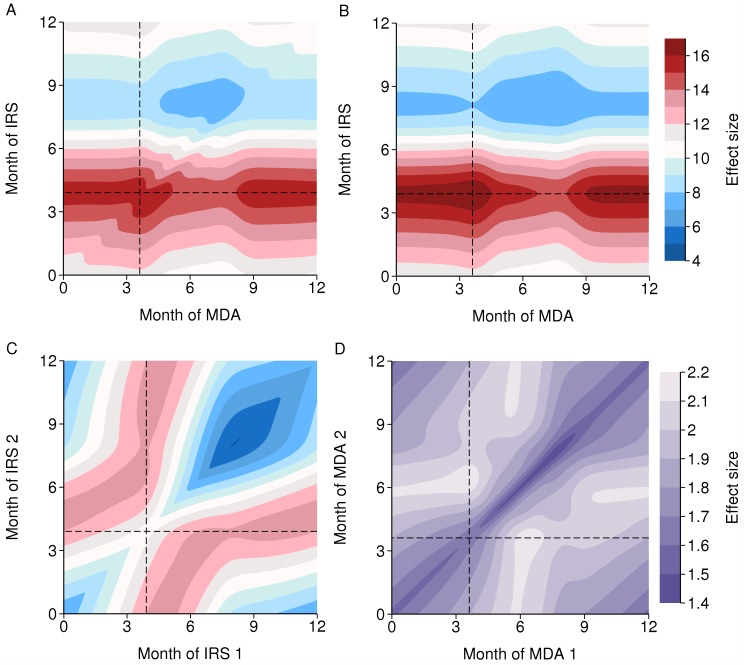
Effect size of two intervention rounds per year. The dashed horizontal and vertical lines mark the optimal time of year for each intervention on its own. There is a 3 month high season centred at 6 months. A: Effect size of a single annual round each of IRS and MDA. B: Product of separate IRS and MDA effect sizes. C: Effect size of two rounds of IRS per year (same colour scheme as A and B). D: Effect size of two rounds of MDA per year.

With seasonality and one round each of IRS and MDA per year, the optimal timing relative to the transmission season is dominated by the optimal timing of each intervention on its own, particularly IRS, as this is more effective than MDA (Fig. 7A). Again, the combined effect size is similar to the product of the separate effect sizes (Fig. 7B), differing by up to 5%, implying only a modest interaction between the two interventions according to their timing.

If there are two rounds of IRS per year with no MDA, then it is best to have one round just before the high season and the other during or after the high season, and the rounds at least three months apart when efficacy decays with a six month half-life (Fig. 7C). One round each of IRS and MDA is more effective than two rounds of IRS, despite a single round of IRS being much more effective than MDA: with a six month half-life of IRS efficacy, there are rapidly diminishing returns from additional rounds per year. Note that these results assume that with repeated rounds of an intervention, the same people at each round are reached, whereas receipt of different interventions are independent of each other, and so the synergy between different interventions is greater than between more rounds of the same intervention for that reason as well. With two rounds of MDA but no IRS, it is best to have one round during the low season and the other just before the peak of the high season, to maximise both effects, of clearing infections and of prophylaxis (Fig. 7D and Fig. 5B).

### Effect size for other interventions

**Figure 8:**
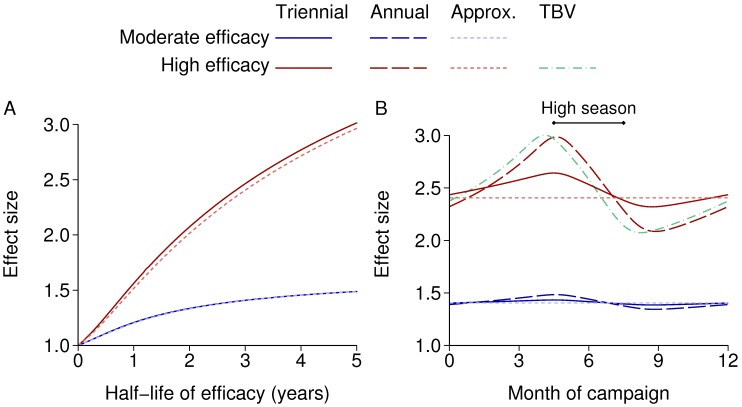
Effect size of a pre-erythrocytic vaccine. The lines labelled “Approx.” are approximate effect sizes calculated using the mean efficacy over the cycle between vaccination campaigns. The lines labelled “Moderate efficacy” have 50% initial efficacy and 80% coverage, while “High efficacy” means 90% initial efficacy and 90% coverage. A: Vaccination every three years (triennial) in a non-seasonal setting, with varying duration of protection. B: Annual vaccination with a one year half-life or triennial vaccination with a three year half-life (so the mean efficacy is the same in both cases), with a three-month high season and varying time of vaccination. Annual vaccination with a high efficacy transmission-blocking vaccine (TBV) is also shown.

Fig. 8 shows the effect size of repeated mass campaigns using a pre-erythrocytic vaccine (PEV) (i.e. preventing initial human infection) with exponentially decaying efficacy. The vaccine is assumed to either have 50% initial efficacy and 80% coverage or 90% initial efficacy and 90% coverage, labelled as moderate and high efficacy respectively. Also shown are approximate effect sizes calculated using the mean efficacy over the cycle between vaccination campaigns. In a non-seasonal setting, the effect size with moderate efficacy is almost exactly the same as the approximate formula, whereas with high efficacy the true effect size is up to 3% larger than that calculated using the mean efficacy. With seasonality, it is best to vaccinate just before the high season. The relative difference that this timing makes is reduced when the duration of protection is longer than one year, or when the vaccine does not have high maximum efficacy.

Annual vaccination with a high efficacy transmission blocking vaccine (TBV) is also shown (Fig. 8B): these reduce transmission from humans to mosquitoes. The interaction with seasonality is similar to the PEV, but with an optimal time 15 days earlier. During the low season the parasite reservoir is mainly in the human population, and so once mosquito numbers increase, transmission is initially from human to mosquito. This step is affected by the TBV, but it is the next step, from mosquito to human, that is affected by the PEV. This explains the difference in optimal timing, because in this model the mean mosquito to human generation time is around 17 days.

The principal intervention against malaria in Africa is currently insecticide-treated nets (ITNs). As they have long-lasting efficacy, and also they are distributed through multiple channels rather than purely in mass campaigns, the population-level efficacy will not change as sharply over one season as with IRS. Hence the following results assume that ITN coverage and efficacy are constant. Even so, the effect size may be different in a seasonal compared to a non-seasonal setting because the duration of infectiousness of the mosquito is shortened by mortality due to ITNs, and this duration interacts with seasonality in its effect on the reproduction number. The effect size without seasonality can be calculated using the reproduction number formulae in the Methods section. In this case, the numerical methods used for the time-varying results gave the same effect size as using reproduction number formulae, to four significant figures.

High efficacy means here that 56% of mosquitoes trying to feed on a protected person are repelled, 41% are killed and the remaining 3% successfully feed. These are the parameters for Anopheles gambiae ss and new nets from [7]. Moderate efficacy means that 44% are repelled and 25% are killed. In the results here, the efficacy does not vary over time, but the moderate efficacy values are taken from the mean efficacies over four years, allowing for decay of insecticidal effect and damage to nets with a half-life of 2.6 years when using the parameters from [7] – in that paper the efficacy did vary over time.

With seasonality and 80% coverage, the effect size is within 2% of its non-seasonal value if the high season is three months or more long, but up to 7.5% different with a two month high season (Table 1). Seasonality may either increase or decrease the effect size.

**Table 1:** Effect size of ITNs with 80% coverage.

**Efficacy**	**Length of high season (months)**	**Effect size**	**Ratio of seasonal and non-seasonal effect sizes**
Moderate	Non-seasonal	6.824	
	2	7.174	1.051
	3	6.845	1.003
	4	6.759	0.990
High	Non-seasonal	21.95	
	2	23.58	1.075
	3	21.98	1.002
	4	21.60	0.984

With a three month high season, the combined effect size of ITNs with either MDA or a pre-erythrocytic vaccine, or MDA with a vaccine, is usually within 1.5% of the product of the separate effects (Table 2). However when a high efficacy annual vaccine is combined with MDA, the combined effect size is 4% less than the product of separate effects.

**Table 2:** Effect size of combinations of interventions.

**Intervention 1**	**Intervention 2**	**Effect size 1**	**Effect size 2**	**Product of effect sizes**	**Effect size of combination**	**Ratio of combination to product**
ITN, moderate	MDA, low season	6.845	1.594	10.91	10.98	1.006
efficacy	MDA, at peak	6.845	1.459	9.99	10.08	1.009
	Vaccine, mod. triennial	6.845	1.433	9.81	9.81	1.001
	Vaccine, mod. annual	6.845	1.484	10.16	10.18	1.002
	Vaccine, high triennial	6.845	2.642	18.09	18.11	1.001
	Vaccine, high annual	6.845	2.985	20.44	20.62	1.009
ITN, high	MDA, low season	21.98	1.594	35.04	35.33	1.008
efficacy	MDA, at peak	21.98	1.459	32.07	32.53	1.014
	Vaccine, mod. triennial	21.98	1.433	31.50	31.52	1.001
	Vaccine, mod. annual	21.98	1.484	32.62	32.71	1.003
	Vaccine, high triennial	21.98	2.642	58.08	58.19	1.002
	Vaccine, high annual	21.98	2.985	65.62	66.44	1.012
MDA, low	Vaccine, mod. triennial	1.594	1.433	2.284	2.276	0.996
season	Vaccine, mod. annual	1.594	1.484	2.365	2.339	0.989
	Vaccine, high triennial	1.594	2.642	4.211	4.156	0.987
	Vaccine, high annual	1.594	2.985	4.758	4.579	0.962

Low season MDA means three months before the peak of the season. “Vaccine” refers to a pre-erythrocytic vaccine six weeks before the seasonal peak, “mod” and “high” refer to a moderate or high efficacy vaccine, and annual and triennial vaccines have one and three year half-lives respectively. There is a three month high season, and interventions are at 80% coverage except the high efficacy vaccine which is 90%.

## Discussion

In a non-seasonal setting when the intervention effects do not vary with time, interventions which target different stages of the transmission cycle have a multiplicative effect on *R*_0_, as long as when there is less than 100% coverage, receipt of each intervention is independent of the others. For the examples considered, the same is approximately true of time-varying control measures, both with and without seasonal variation, and hence simple assessments of combined effect sizes are possible once the individual effect sizes have been calculated. However, it will often be the same people who receive different interventions, particularly if they are delivered by a single programme. In that case we would in general expect the combined effect size to be less than the product of the separate effect sizes.

When there is seasonality, the effect size of individual time-varying interventions can vary greatly according to what time of year they are implemented, and it is necessary to use methods such as those described here rather than plugging the average efficacy into a simple formula. For both IRS and MDA, the effect size increases with increasing seasonality if the intervention is carried out at the optimal time of year. Even without seasonality, the true effect size of time-varying interventions can be substantially different from that calculated using the mean efficacy or the mean of a reproduction number formula over the cycle between rounds, particularly for an intervention such as IRS with high maximum efficacy but with a duration of effect which is short relative to the period between spraying rounds. For maximum effect size, the optimal time of year for IRS, a vaccine or any other prophylactic measure is just before the high season, whereas the best time to clear the reservoir of human infection using MDA is any time during the low season.

The focus in this paper has been on *R*_0_, but the burden of disease and mortality are the most important aspects of malaria. If we are assessing interventions that even in combination are unable to locally eliminate malaria, then we should consider the effect on morbidity and mortality, including by using simulation models which include immunity in a realistic way. In such cases, the effect size still provides a measure of the reduction in transmission achievable by an intervention, which will translate into a changed burden of disease. However this change will not in general be proportional to the effect size. Moreover a given effect size may correspond to different reductions in morbidity and mortality depending on the intervention, even for the same pre-intervention conditions: for example scaling up treatment coverage may directly reduce progression to severe malaria and death as well as reducing the reproduction number and the incidence of new infections, and so could have a greater impact on mortality than a different intervention with the same effect size.

If elimination is the goal, then *R*_0_ needs to be reduced to below 1 everywhere, and the effect size as used here is the most direct quantification of how much an intervention contributes to this. Furthermore, the effect size is a single summary of impact which is independent of the baseline transmission intensity. The relative or absolute changes in other outcomes such as EIR, parasite prevalence and incidence of disease do depend on transmission intensity, and also on the time horizon over which they are measured from when the intervention is introduced, and on acquired immunity, particularly how fast immunity is lost once transmission is reduced. It may be that immunity reduces the reproduction number below *R*_0_ and so a smaller effect size would be needed to locally eliminate, if this could be done before population-level immunity has substantially waned from its endemic level. On the other hand, in sub-Saharan Africa interventions are being scaled up over many years, and after elimination immunity will eventually disappear, and so *R*_0_ needs to be reduced below 1 unless reintroduction of infection can be prevented.

Previous work using simulation models has already shown that the optimal time for IRS is at the start of the high season, so that efficacy persists over as much of the transmission season as possible [7,8]. It has also been shown that the best time for MSAT (mass screening and treatment) is in the low season. Using different endpoints in each case, the best time was reported as being at start of the period of lowest EIR [7], towards the end of the low season [9], or one month before the trough in EIR [10]. The effect sizes calculated here for IRS and ITNs are smaller than the values of over 100 for ITNs at above 90% coverage reported in [4], as they assume 80% coverage by default and/or allow for a loss of efficacy of insecticide and damage to nets. If a high lethal effect plus high coverage can be maintained then the effect sizes do increase to those higher values for mosquitoes which feed indoors at night, and rest indoors in the case of IRS.

The reproduction numbers and effect sizes presented here do not take account of saturation due to finite population size, which reduces *R*_0_ [11]. However, I would argue that *R*_0_ calculated ignoring finite population effects (the infinite population *R*_0_) is a more useful quantity than the finite population *R*_0_: the saturation effects disappear as *R*_0_ is reduced towards 1, and so the infinite population *R*_0_ (which is proportional to quantities such as infection rates and mosquito density) tells us by how much transmission needs to be reduced for elimination, whereas the finite population *R*_0_ does not. Furthermore, the infinite population *R*_0_ scale is the scale on which the effect size is independent of the baseline transmission intensity.

There has been a renewed focus in recent years on reducing malaria transmission, and even global eradication. In sub-Saharan Africa, *R*_0_ due to *Plasmodium falciparum* malaria has been estimated at over 1000 when there is intense transmission [11]. So in order to eliminate the infection it is necessary to combine multiple interventions and to use each one optimally. The methods and results described here will help to guide this process, complementing simulation models. Calculating the effect on *R*_0_ of different interventions puts them on a common scale so that they can be compared in their ability to reduce transmission. In particular there has been a revival of interest in various forms of mass treatment for malaria [12]. There has not previously been an assessment of the effect size of these interventions, although simulation models have looked at other endpoints.

Many diseases have seasonally varying transmission, particularly vector-borne infections with vector numbers varying due to rainfall or temperature. In other cases there is seasonality due to school holidays or survival of pathogens in the environment. Many of the results derived in the methods section apply directly to other diseases, as they do not depend on specific features of the malaria model considered. Heterogeneity in human exposure to disease vectors will increase *R*_0_ by the same factor with seasonality as it does without seasonality for vector-borne diseases in general, and the same holds for the increase in *R*_0_ due to heterogeneity in contact rates for directly transmitted pathogens. If there is variation between people or other hosts in the time-course or magnitude of onwards infectiousness, it is only necessary to consider the mean infectious profile, even when there is seasonal variation. Also, for interventions with constant effectiveness over time, the effect size in reducing *R*_0_ is the same with or without seasonality, as long as the time course of expected infectivity is not changed.

## Methods

### Calculation of R_0_ from next generation matrix

The basic reproduction number *R*_0_ is defined as the average number of infections resulting from one typical case in an otherwise susceptible population. For a malaria model in which humans and mosquitoes are homogeneous, it is possible to write down a simple formula for *R*_0_ for human-to-human transmission (via one mosquito generation). When there are multiple types of possible human or mosquito hosts in a population, then simply averaging the number of secondary cases in proportion to the occurrence of the types of primary hosts in the population will in general be incorrect if the number of onward infections an individual produces is correlated with the probability of becoming infected: the quantity calculated in this way may not have the threshold property that large outbreaks are possible if and only if it is above 1. In a non-seasonal environment, *R*_0_ can be correctly calculated from the next generation matrix as described in [13]. In this approach, infected hosts are divided into types defined by their “state at infection”, which consist of all possible states relevant to onward transmission that a host can be in immediately after infection. The entry in the next generation matrix *K_ij_* is the expected number of infections of type *i* that result over the course of an infection of type *j*.

If mosquitoes and humans are divided into *n_M_* and *n_H_* categories respectively with different characteristics, and if humans and mosquitoes are considered as separate generations, then *K* takes the form

$$K=\left[\begin{array}{cc} 0_{n_{H}\times n_{H}} & K^{HM}\\ K^{MH} & 0_{n_{M}\times n_{M}} \end{array}\right]$$ (3)

where *K^MH^* is an *n_M_* × *n_H_* matrix whose element *K^MH^_ij_* is the expected number of mosquito infections of type *i* resulting from an infected human of type *j*, and similarly for the *n_H_* × *n_M_* matrix *K^HM^* for mosquito to human transmission. 0_*n×n*_ denotes an *n×n* matrix of zeroes.

Throughout this paper the reproduction number is defined as being for mosquito-to-mosquito (or equivalently human-to-human) transmission. The expected number of mosquitoes of type *i* infected by an infected mosquito of type *k* via a single human generation can be found by summing over the possible types of intermediate human:

$$K^{MM}{}_{ik}=\sum_{j=1}^{n_{H}}K^{MH}{}_{ij}K^{HM}{}_{jk}$$

Hence the next generation matrix for mosquito-to-mosquito transmission is

$$K^{MM}=K^{MH}K^{HM}$$

and *R*_0_ is the leading eigenvalue of this matrix.

If heterogeneity in host characteristics is indexed by continuous parameters in one or more dimensions instead of a finite number of categories, then *R*_0_ can be defined in terms of the spectral radius of a next generation operator, but the matrix representation is used here as it will be more familiar to most readers.

### Definition of R_0_ in a periodic environment

In what follows, the periodic system is described in terms of years for simplicity, although it applies to a period of any length. The key insight of Bacaër and co-authors is that in a seasonally varying environment, the time of year that a host becomes infected can be considered as another aspect of their state at infection. This insight gives a clear interpretation that was previously lacking of what the reproduction number means in a periodic setting. Bacaër [1] derives several methods for calculating the reproduction number with seasonal variation in transmission. Two of these are generally applicable numerical methods and are described here. The first is based on directly considering the next generation matrix, and is the method used for the results in this paper. The second is based on numerically solving the model differential equations.

The first method of calculating the reproduction number is to divide the year into intervals which are small enough that conditions are approximately constant within the interval, and have separate entries in the next generation matrix for humans and mosquitoes infected at each time of year. Suppose first that humans and mosquitoes are homogeneous apart from seasonal variation, and the year of length *T* is divided into *N* intervals of length δ = *T*/*N*, so that *i* represents the time of year (*i* – 1)*δ* to *iδ* for *i* = 1,…,*N*. In the notation of equation (3)

$$\begin{array}{l} K_{T}^{MH}{}_{ij}=m(i\delta )\alpha (i\delta )\delta \left[I_{i>j}\rho \left((i-j)\delta ,i\delta \right)+\sum_{v=1}^{\infty }\rho \left((i-j)\delta +vT,i\delta \right)\right]\\ K_{T}^{HM}{}_{jk}=\alpha (j\delta )\delta \left[I_{j>k}\sigma \left((j-k)\delta ,j\delta \right)+\sum_{v=1}^{\infty }\sigma \left((j-k)\delta +vT,j\delta \right)\right]\\ i,j,k=1,...,N \end{array}$$ (4)

$K_{T}^{MH}{}_{ij}$ is the expected number of mosquitoes infected at the time of year indexed by *i* by a human who was infected at the time of year indexed by *j*. Interval *i* can be in the same year as interval *j* if *i* > *j*, which is why the indicator function *I*_*i*>*j*_ appears, or it can be in any future year, which is why the sum $\sum_{v=1}^{\infty }$ appears. $K_{T}^{HM}{}_{jk}$ is the analogous term for mosquito to human transmission.

*ρ*(*u*,*t*) and *σ*(*u*,*t*) are the expected infectivity of humans and mosquitoes respectively at time *u* after infection and at time of year *t*, *m*(*t*) is the mosquito density per person and *α*(*t*) is the rate at which mosquitoes bite humans. This formulation can include interventions with time-varying effects that are implemented in a repeated periodic pattern, so that one or more of *ρ*(*u*,*t*), *σ*(*u*,*t*), *m*(*t*) and *α*(*t*) vary periodically due to the intervention(s).

The next generation matrix for mosquito-to-mosquito transmission is $K_{T}^{MM}=K_{T}^{MH}K_{T}^{HM}$. Suppose that *m*(*t*) is replaced by *θ* *m*(*t*) for all 0 ≤ *t* < *T*, for some constant positive factor *θ*. Then $K_{T}^{MH}$ is replaced by $\theta K_{T}^{MH}$, and $K_{T}^{MM}$ is replaced by $\theta K_{T}^{MM}$. So *R*_0_, which is the leading eigenvalue of $K_{T}^{MM}$, is multiplied by *θ*. This result will be used in the second method for calculating *R*_0_.

### Heterogeneity in human biting rates

As well as variation over time, the human population may also be divided into subgroups with differing characteristics relevant to transmission. One important type of variation is that some people are bitten by mosquitoes more often than others. Suppose that people are divided into *n_ζ_* groups, with a proportion *p_h_* in group *h* who are bitten at rate *ζ_h_* *α*(*t*) at time of year *t*, where $\sum_{h=1}^{n_{\zeta }}p_{h}\zeta_{h}=1$. A continuous distribution of biting rates may be approximated in this way.

The next-generation matrix with humans indexed both by time of year and biting rate is

$$K_{\zeta }=\left[\begin{array}{cc} 0_{n_{\zeta }N\times n_{\zeta }N} & K_{\zeta }{}^{HM}\\ K_{\zeta }{}^{MH} & 0_{N\times N} \end{array}\right]=\left[\begin{array}{cc} 0_{n_{\zeta }N\times n_{\zeta }N} & \begin{array}{c} p_{\zeta 1}\zeta_{1}K_{T}^{HM}\\ p_{\zeta 2}\zeta_{2}K_{T}^{HM}\\ \vdots \\ p_{\zeta n_{\zeta }}\zeta_{n_{\zeta }}K_{T}^{HM} \end{array}\\ \begin{array}{cccc} \zeta_{1}K_{T}^{MH} & \zeta_{2}K_{T}^{MH} & \cdots & \zeta_{n_{\zeta }}K_{T}^{MH} \end{array} & 0_{N\times N} \end{array}\right]$$

where the matrices $K_{T}^{MH}$ and $K_{T}^{HM}$ are as defined in equation (4).

*R*_0_ is the leading eigenvalue of

$$K_{\zeta }{}^{MH}K_{\zeta }{}^{HM}=\left(\sum_{h=1}^{n_{\zeta }}p_{\zeta h}\zeta_{h}{}^{2}\right)K_{T}^{MH}K_{T}^{HM}=\left(1+c_{v}{\left(\zeta \right)}^{2}\right)K_{T}^{MH}K_{T}^{HM}$$

where *c_v_*(*ζ*) is the coefficient of variation of *ζ*, the relative biting rate. Hence *R*_0_ is multiplied by a factor of 1 + *c_v_*(*ζ*)^2^ compared to its value with no heterogeneity in biting between people, just as in the time-invariant case [14].

This result may be generalised to the case where humans are grouped by other factors in addition to time of year and biting rate, if heterogeneity in biting is independent of the other factors, and the other factors can include groups who do and do not receive an intervention. The reproduction numbers with and without an intervention are both multiplied by 1 + *c_v_*(*ζ*)^2^, as long receipt of the intervention is not targeted at or misses those who are bitten most, and so the effect size is the same as it would be without heterogeneity in biting.

### Heterogeneity in human infectious profiles

There may also be heterogeneity in both the time course and overall extent of human to mosquito infectiousness. Suppose that there are *n_ρ_* groups, with a proportion *p_ρh_* in group in group *h* whose infectiousness to mosquitoes is *ρ_h_*(*u,t*) at time *u* after infection and at time of year *t*. The next generation matrix is

$$K_{\rho }=\left[\begin{array}{cc} 0_{n_{\rho }N\times n_{\rho }N} & K_{\rho }{}^{HM}\\ K_{\rho }{}^{MH} & 0_{N\times N} \end{array}\right]=\left[\begin{array}{cc} 0_{n_{\rho }N\times n_{\rho }N} & \begin{array}{c} p_{\rho 1}K_{T}^{HM}\\ p_{\rho 2}K_{T}^{HM}\\ \vdots \\ p_{\rho n_{\rho }}K_{T}^{HM} \end{array}\\ \begin{array}{cccc} K_{\rho 1}^{MH} & K_{\rho 2}^{MH} & \cdots & K_{\rho n_{\rho }}^{MH} \end{array} & 0_{N\times N} \end{array}\right]$$

with, using the notation of equation (4), each $K_{\rho h}^{MH}$ having entries

$$\begin{array}{l} K_{\rho h}^{MH}{}_{ij}=m(i\delta )\alpha (i\delta )\delta \left[I_{i>j}\rho_{h}\left((i-j)\delta ,i\delta \right)+\sum_{v=1}^{\infty }\rho_{h}\left((i-j)\delta +vT,i\delta \right)\right]\\ h=1,...,n_{\rho };\;i=1,...,N;\;j=1,...,N \end{array}$$

*R*_0_ is the leading eigenvalue of

$$K_{\rho }{}^{MH}K_{\rho }{}^{HM}=\left(\sum_{h=1}^{n_{\rho }}p_{\rho h}K_{\rho h}^{MH}\right)K_{T}^{HM}=K_{T}^{MH}K_{T}^{HM}$$

where $K_{T}^{MH}$ is as defined in equation (4), with $\rho (u,t)=\sum_{h=1}^{n_{\rho }}p_{\rho h}\rho_{h}(u,t)$.

So when finding *R*_0_, it is only necessary to consider the mean infectious profile over time and not its variation between people, even when there is seasonal variation. This formulation allows *ρ_h_*(*u,t*) to depend on the time of year as well as time since infection, and so if there is repeated periodic mass drug administration that clears some infections, we can still just use the mean infectious profile to find the reproduction number with the intervention.

### Time-invariant interventions

Consider a pre-erythrocytic vaccine that covers a proportion *p_V_* of the human population and has efficacy *e_V_*, so that their probability of becoming infected is multiplied by 1 – *e_V_*, with negligible waning of efficacy. The next-generation matrix is

$$K_{V}=\left[\begin{array}{cc} 0_{2N\times 2N} & K_{V}{}^{HM}\\ K_{V}{}^{MH} & 0_{N\times N} \end{array}\right]=\left[\begin{array}{cc} 0_{2N\times 2N} & \begin{array}{c} p_{V}(1-e_{V})K_{T}^{HM}\\ (1-p_{V})K_{T}^{HM} \end{array}\\ \begin{array}{cc} K_{T}^{MH} & K_{T}^{MH} \end{array} & 0_{N\times N} \end{array}\right]$$

with $K_{T}^{MH}$ and $K_{T}^{HM}$ as defined in equation (4).

The reproduction number is the leading eigenvalue of

$$K_{V}{}^{MH}K_{V}{}^{HM}=\left(p_{V}(1-e_{V})+(1-p_{V})\right)K_{T}^{MH}K_{T}^{HM}$$

Hence the reproduction number with the vaccine is

$$R_{C}=\left(p_{V}(1-e_{V})+(1-p_{V})\right)R_{0}=\left(1-p_{V}e_{V}\right)R_{0}$$

The effect size of the vaccine is *R_0_*/*R_C_* = 1/(1−*p_V_e_V_*), the same as in a non-seasonal setting.

Similarly suppose that a transmission-blocking vaccine covers a proportion *p_TBV_* of the human population and has efficacy *e_TBV_* at reducing human infectiousness to mosquitoes. The next-generation matrix is

$$K_{TBV}=\left[\begin{array}{cc} 0_{2N\times 2N} & K_{TBV}{}^{HM}\\ K_{TBV}{}^{MH} & 0_{N\times N} \end{array}\right]=\left[\begin{array}{cc} 0_{2N\times 2N} & \begin{array}{c} p_{TBV}K_{T}^{HM}\\ (1-p_{TBV})K_{T}^{HM} \end{array}\\ \begin{array}{cc} (1-e_{TBV})K_{T}^{MH} & K_{T}^{MH} \end{array} & 0_{N\times N} \end{array}\right]$$

The reproduction number is the leading eigenvalue of

$$\begin{array}{c} K_{TBV}{}^{MH}K_{TBV}{}^{HM}=\left(p_{TBV}(1-e_{TBV})+(1-p_{TBV})\right)K_{T}^{MH}K_{T}^{HM}\\ =\left(1-p_{TBV}e_{TBV}\right)K_{T}^{MH}K_{T}^{HM} \end{array}$$

and so the effect size is 1/(1−*p_TBV_e_TBV_*), again the same as in a non-seasonal setting.

By analogous arguments this holds for any intervention which changes infection rates by a constant factor, from mosquitoes to humans, vice versa, or both, but does not change the time course of expected infectivity from the point of infection onwards, and whose coverage and efficacy do not change over time. The same is true of interventions which change the biting rate on humans by a constant factor, such as a non-lethal repellent or nets which have not been treated with insecticide.

On the other hand, if there is an increase in the mosquito death rate, as with ITNs, then the duration of infectiousness of the mosquito is reduced. Hence the infectious profile over time since infection is changed, and so the effect size will in general be different to what it is in a non-seasonal setting even if the coverage and efficacy are constant.

### Floquet theory

A second general method was also introduced in [1] to calculate the reproduction number, based on Floquet theory, which is a branch of mathematics that deals with periodic differential equations. Suppose that the transmission model can be represented as a set of ordinary differential equations with periodic coefficients, of period *T*. Let *Y* be the *n*×1 vector of all infected states. The linearised version of the model has the form:

$$\frac{dY(t)}{dt}=\Lambda (t)Y(t)$$ (5)

where Λ(*t*) is an *n*×*n* periodic matrix, Λ(*t* + *T*) = Λ(*t*) for all *t*.

Floquet theory shows that the monodromy matrix *M* associated with this system has the property that the infection-free state is unstable if and only if the largest eigenvalue of *M* is greater than one [1].

*M* can be found by filling it in one column at a time. For *i* = 1 to *n*:

- set element *i* of *Y*(0) to 1 and the other entries to 0;
- numerically solve the model of equation (5) from *t* = 0 to *T*;
- set column *i* of *M* equal to *Y*(*T*).

Recall that if the possibly time-varying mosquito density *m*(*t*) is multiplied by a constant positive factor *θ*, then *R*_0_ is also multiplied by *θ*. This allows us to calculate *R*_0_. The procedure is to use an iterative root-finding algorithm to find the value of *θ* for which the monodromy matrix *M* has largest eigenvalue equal to 1, with the original *m*(*t*) replaced by *θm*(*t*) for all 0 ≤ *t* ≤ *T* at each iteration. Denote the value of *θ* found by the root-finding as *θ*_0_. Since both thresholds, of *R*_0_ and the largest eigenvalue of *M* being 1, determine the stability of the infection-free state, they must coincide. Hence the reproduction number is 1 when *m*(*t*) is replaced by *θ*_0_ *m*(*t*), and so the reproduction number with the original *m*(*t*) is *R*_0_ = 1/*θ*_0_.

The two methods for calculating *R*_0_, either discretising the time of year and finding the largest eigenvalue of the next generation matrix or using the approach based on Floquet theory, give the same numerical value as long as in the former method, the year is divided up sufficiently finely. Also, it is shown in [15] that this *R*_0_ has the same interpretation as in the non-seasonal case, as a long run per-generation growth rate in the incidence of new infections. The 2012 edition of one of the standard textbooks on infectious disease dynamics has a summary of the approach introduced by Bacaër and others, recognising it as the correct way to calculate the reproduction number in periodic conditions [13].

### Effect size of time-varying interventions

Bacaër and co-authors developed the methods described in the previous sections and applied them to diseases where transmission has natural seasonal variation [1,2]. However transmission may also vary over time due to interventions. If interventions are applied in a periodic pattern that repeats indefinitely, then the definition of *R*_0_ in a seasonal environment can be extended to cover this case. Hence to calculate the effect size of time-varying interventions, I used the next generation matrix method to find both *R*_0_ and *R_C_*, the reproduction number with one or more interventions in place, and the effect size *E* is the ratio of these

$$E=\frac{R_{0}}{R_{C}}$$

The reason for using the next generation matrix method is that it is more suitable for a model where infectiousness is defined by time since infection, as is the case here for human infectiousness to mosquitoes. A large number (*n*) of model states are needed to closely approximate the infectious profile over time, and the computing time for the Floquet theory method increases in proportion to *n* ^2^, a factor of *n* for solving the model over one cycle and another factor of *n* for the number of columns in the monodromy matrix. However, the Floquet theory method will often be easier to implement, particularly if one has already coded the model differential equations. I also calculated results for each intervention considered using the Floquet theory method as a check for correctness, and they were similar to the next generation matrix method, becoming closer as more states are used to approximate the infectious profile in the Floquet theory method.

To find the reproduction number by discretising the year into *N* intervals, I filled in the matrix *K_MM_*, and then used the power iteration method [16] to find the largest eigenvalue. As the overall computing time increases in proportion to at least *N* ^2^, I used extrapolation to obtain accurate results with moderate *N*. If we consider the calculated reproduction number *R*(*δ*) as a function of the width of the intervals *δ* = 1/*N* into which the year is divided, then we can linearly extrapolate to *δ* = 0 as follows for any two positive values of *δ*:

$$R(0)=\frac{R(\delta_{2})\delta_{1}-R(\delta_{1})\delta_{2}}{\delta_{1}-\delta_{2}}$$

For example, with $\delta_{1}=\frac{1}{N}$ and $\delta_{2}=\frac{1}{2N}$, we have

$$R(0)=\frac{R\left(\frac{1}{2N}\right)\frac{1}{N}-R\left(\frac{1}{N}\right)\frac{1}{2N}}{\frac{1}{N}-\frac{1}{2N}}=2R\left(\frac{1}{2N}\right)-R\left(\frac{1}{N}\right)$$

Using the pair *N* = 2 × 365, 4 × 365 generally gave results that were accurate to at least three significant figures.

### Seasonal curves

For time units of one year (*T* = 1), the functional form considered for the seasonally varying mosquito density *m*(*t*) at time *t* is

$$\begin{array}{l} m(t)=m_{0}\left(c+(1-c)g(t)\right),\;0<c\leq 1\\ g(t)=\frac{1}{\gamma }{\left(\frac{1+\cos\left(2\pi (t-t_{0})\right)}{2}\right)}^{\kappa },\kappa >0 \end{array}$$ (6)

*γ* is a normalising constant chosen so that *g*(*t*) has a mean over the year of 1, and hence *m*(*t*) has a mean of *m*_0_

$$\gamma =\int_{0}^{1}{\left(\frac{1+\cos\left(2\pi (t-t_{0})\right)}{2}\right)}^{\kappa }\;dt=\frac{B\left(\frac{1}{2},\kappa +\frac{1}{2}\right)}{\pi }$$

where *B* is the Beta function. For ease of interpretation, *m*(*t*) is parameterised in terms of *d* instead of *κ*, where *d* is the duration of the high season, defined as the period with *m*(*t*) ≥ *m*_0_. For a given *d*, the implied value of *κ* is found numerically. When *d* is equal to six months, then *κ* = 1 and the curve is sinusoidal of the form considered by Bacaër in [1].

**Figure 9:**
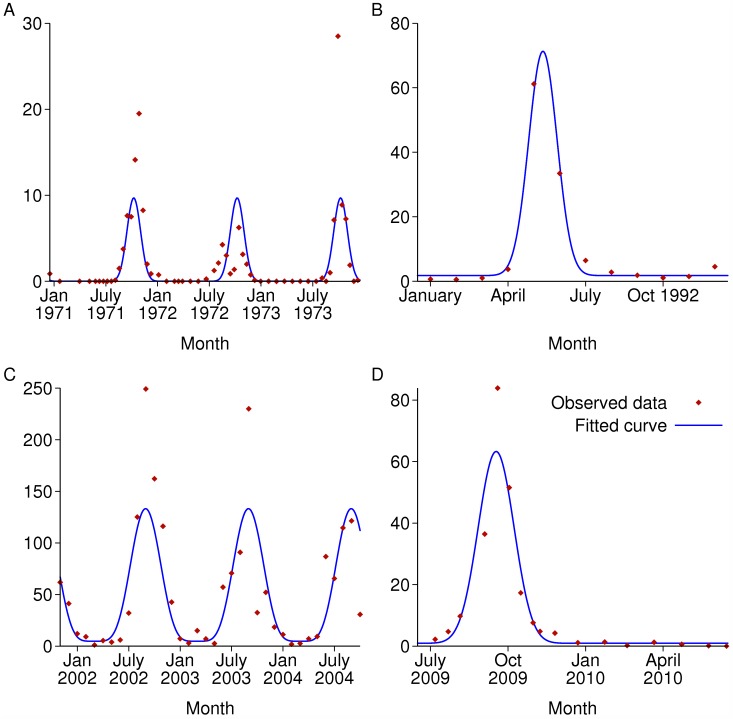
Recorded seasonally varying mosquito densities from published papers and fitted curves. As the sampling methods and other factors vary between the datasets, the units for the data are not comparable between sites and have been omitted. A: Ajura village, Garki, Nigeria; B: Bagamayo, Tanzania; C: Navrongo, Ghana; D: Dakar, Senegal.

The parametric form of equation (6) was fitted to four published datasets of seasonally varying mosquito densities. The fitting method was to minimise the sum of squares of the difference between the square root of the data and the square root of the predicted value. For simplicity each dataset is the sum of all Anopheles species reported. As the sampling methods and other factors vary between the datasets, the units for the data are not comparable between sites and have been omitted. The fitted parameters *c* and *d*, which determine the shape of the curve, are given in Table 3, and the observed and fitted curves are shown in Fig. 9. *c* is in general difficult to estimate precisely, but *d* is more precisely estimated. In the subsequent results, to represent a highly seasonal setting the default values used were 3 months for *d* and 0.05 for *c*, with other values also explored.

**Table 3:** Parameters for curves fitted to mosquito density data with 95% confidence intervals.

Site, with reference	Width of high season, months (*d*)		Low season relative to mean (*c*)	
	Estimate	95% CI	Estimate	95% CI
Ajura, Garki, Nigeria [22]	3.0	2.5, 3.4	0.004	0.0004, 0.035
Bagamayo, Tanzania [23]	2.3	1.9, 2.7	0.18	0.10, 0.29
Navrongo, Ghana [24]	4.9	4.2, 5.5	0.10	0.05, 0.18
Dakar, Senegal [25]	2.7	2.4, 3.0	0.10	0.04, 0.20

### Malaria model

The effect sizes calculated in this paper are for populations with no acquired immunity to malaria. Human infectiousness to mosquitoes as a function of time since infection was based on a recently published within-host model of malaria [17]. The authors parameterised the model by re-analysing malaria-therapy data collected from patients who were infected with *Plasmodium falciparum* malaria as a treatment for syphilis, and who also participated in mosquito feeding studies. A program implementing the model of [17] was published along with the paper, which I used to generate 40,000 infectious profiles, and then calculated the mean infectiousness on each day.

The results here do not include any treatment for symptomatic malaria. If some infections are treated and cleared during the infectious period, then the overall time-course of human infectiousness is modified and the effect size of time-varying interventions will be altered. However if treatment for clinical malaria takes place early enough after blood-stage infection appears, particularly with a drug such as an artemisinin combination therapy (ACT) which can kill the sexual stages of the parasite, then almost all onward transmission would result from those who are not treated or who are asymptomatically infected. This means that the reproduction number would be reduced by treatment, but that the effect size of other interventions and the impact of seasonality on the reproduction number would be less affected.

The parameters for the mosquito life cycle and for the effect of interventions targeting mosquitoes were set at the values used for *Anopheles gambiae ss* in [7].

### Intervention models

For all interventions with repeated mass campaigns, it is assumed that the same people are covered at each round, but that different interventions are independent of each other in the choice of who is covered.

### MDA

MDA is assumed to clear existing infections after the first 10 days, representing the liver stage, and also to provide prophylaxis against new infections with an exponentially distributed duration of protection with a mean of 30 days, based on the long elimination half-lives of drugs such as piperaquine and amodiaquine [18]. Two other durations of prophylaxis are also considered: 10 days and 60 days, the latter being longer than currently available drugs provide.

Suppose that MDA takes place every year at time *t_M_*, and that there is a delay of length *d_E_* between infection and parasites appearing in the blood. Using the notation of equation (4), clearance of existing infections means that for those receiving MDA, onward infectiousness at time *u* after infection for someone infected at time *t* is

*ρ*_*M*_(*u,t*) = 0 if *u* > *T* + *d_E_*, *t* + *d_E_* ≤ *t_M_* < *t* + *u* or *t* + *d_E_* ≤ *t_M_* + *T* < *t* + *u*

*ρ_M_*(*u,t*) = *ρ*(*u,t*) otherwise.

Hence the total onwards infectiousness is lower than it would be without MDA. The delay from clearing asexual parasites to reduction in gametocytes (the sexual stage of the parasite) is not modelled.

Prophylaxis of mean duration *d_P_* prevents the emergence of infection to the blood stage, so MDA at time *t_M_* can be modelled as multiplying the probability of initial infection at time *t* by a factor of 1 − exp(−((*t*+*d_E_*−*t_M_*) mod *T*)/*d_P_*).

### ITNs and IRS

IRS and ITNs are modelled as detailed in [7], with one addition, which was to incorporate the reduced emergence rate of adult mosquitoes that results when the adult mosquito population is reduced, using the model of larval dynamics and parameters described in [19]. The inclusion of this larval model results in a modest increase in the efficacy of lethal interventions that have high coverage compared to a model that assumes the adult mosquito emergence rate is not affected by interventions.

In the larval model, the mosquito density is determined by the carrying capacity of the environment *A*(*t*), which is unaffected by the interventions considered here, although it could be reduced by measures directed at immature mosquito stages. In the absence of interventions and if *A*(*t*) changes smoothly with no sudden step changes, the adult mosquito density *m*(*t*) is almost exactly proportional to *A*(*t*), but with a time lag of 8 days when the parameters from [19] are used. So to have *m*(*t*) follow equation (6), *A*(*t*) takes the same functional form with the same *c* and *κ*, but with the peak time *t*_0_ 8 days earlier.

### Effect size formulae

As in [17], the reproduction number in a time-invariant setting may be expressed as

$$R_{0}=V_{0}D$$

where *V*_0_ is the vectorial capacity and *D* is overall expected human infectiousness to mosquitoes if bitten by an infectious mosquito, summed over the human infectious period. Note that *D* incorporates the probability of mosquito to human infection (but not the probability that a mosquito survives long enough to become infectious) as well as human to mosquito infection.

*D* is unchanged by ITNs, and so for ITNs with constant coverage and efficacy, and with no seasonality, the effect size is the relative change in *V*_0_, similar to the formulae in [20] but with two additions: the human population is explicitly divided into those covered and not covered, and there is an extra term for the reduced emergence rate of adult mosquitoes. With no ITNs, and without heterogeneity between people in how often they are bitten, *R*_0_ is given by

$$R_{0}=\frac{DA\eta (\mu_{0})\alpha_{0}{}^{2}e^{-\mu_{0}\tau }}{\mu_{0}{}^{2}}$$

Here, *Aη*(*μ*_0_) is the emergence rate of adult female mosquitoes in the equilibrium solution to the larval model of [19] with carrying capacity *A* and adult mosquito death rate *μ*_0_. *η*(*μ*_0_) is a function of the larval model parameters as well as *μ*_0_, but is independent of *A*. *Aη*(*μ*_0_)/*μ*_0_ is the mosquito density per person. Mosquitoes bite humans at rate *α*_0_ and have an incubation period of fixed length *τ* before becoming infectious. *α*_0_ can be written as *Q*_0_ *f*_0_, where *Q*_0_ is the proportion of bites that are on humans (anthropophagy) and *f*_0_ is the overall mosquito feeding rate.

Suppose that a proportion *p_C_* of people sleep under a net at night. The reproduction number is now

$$R_{C}=\frac{DA\eta (\mu_{C})\left((1-p_{C})\alpha_{U}{}^{2}+p_{C}\alpha_{C}{}^{2}\right)e^{-\mu_{C}\tau }}{\mu_{C}{}^{2}}$$

where *μ_C_* is the mosquito death rate with the control measure in place, and *α_C_* and *α_U_* are the rates at which mosquitoes successfully bite people who do or do not sleep under nets respectively, using the formulae in [7].

*α_C_* and *α_U_* can be expressed as

$$\begin{array}{l} \alpha_{C}=Q_{C}f_{C}w_{C}/\bar{w}\\ \alpha_{U}=Q_{C}f_{C}/\bar{w}\\ \bar{w}=(1-p_{C})+p_{C}w_{C} \end{array}$$

where *Q_C_* and *f_C_* are the anthropophagy and feeding rate with this net coverage, and *w_C_* is the probability that a feeding attempt on a human who sleeps under a net ends in the mosquito feeding and surviving (averaging over attempts when that person is under and not under their net).

So the reproduction numbers are

$$\begin{array}{l} R_{0}=\frac{DA\eta (\mu_{0})Q_{0}{}^{2}f_{0}{}^{2}e^{-\mu_{0}\tau }}{\mu_{0}{}^{2}}\\ R_{C}=\frac{DA\eta (\mu_{C})Q_{C}{}^{2}f_{C}{}^{2}\left((1-p_{C})+p_{C}w_{C}{}^{2}\right)e^{-\mu_{C}\tau }}{{\bar{w}}^{2}\mu_{C}{}^{2}} \end{array}$$ (7)

The formula for *R_C_* with IRS is the same as for ITNs, except that a mosquito may bite a human and transmit malaria immediately before being killed by IRS, and so with the subscript *C* now meaning control by IRS, we have

$$R_{C}=\frac{DA\eta (\mu_{C})Q_{C}{}^{2}f_{C}{}^{2}\left((1-p_{C})+p_{C}w_{C}y_{C}\right)e^{-\mu_{C}\tau }}{{\bar{w}}^{2}\mu_{C}{}^{2}}$$ (8)

where *y_C_* is the probability that a feeding attempt on a human whose house is protected by IRS ends in a bite on that human, after which the mosquito may or may not survive. Details of how ITNs and IRS affect *μ_C_*, *f_C_*, *Q_C_*, *w_C_* and *y_C_* are given in [7].

### Vaccine

The pre-erythrocytic vaccine is assumed to work by preventing a certain proportion of new infections in humans, with efficacy decaying exponentially over time. A partially effective vaccine which reduces the probability of infection equally for all vaccinees is described as “leaky”, whereas “all-or-nothing” means that a certain proportion of vaccinees are fully protected (before any decay in efficacy), and the rest are not protected. For calculation of the reproduction number, this distinction does not make any difference (for a given mean effective coverage), since we are only considering a single infectious challenge for each person. The approximate effect sizes *E_approx_* in Fig. 8 use the mean efficacy *ē_v_* over the cycle between vaccination campaigns. The formula used is

$$\begin{array}{l} E_{approx}=\frac{1}{(1-p_{V})+p_{V}\left(1-{\bar{e}}_{V}\right)}\\ {\bar{e}}_{V}=(e_{V0}/\delta_{V})\left(1-\exp(-\delta_{V})\right),\;\;\delta_{V}=T\log(2)/d_{V} \end{array}$$ (9)

where *p_V_* is the coverage, *e*_*V*0_ is the initial efficacy, *T* is the time between campaigns and *d_V_* is the half-life of efficacy. Two values for *e*_*V*0_ are considered: 50%, which is similar to that estimated for the RTS,S vaccine in recent phase three trials [21], and a hypothetical vaccine with 90% initial efficacy.

## Supporting Information

S1 Dataset R code [26] that can be used to reproduce the results in this paper. (ZIP)

## References

[1] Bacaër N (2007) Approximation of the basic reproduction number R 0 for vector-borne diseases with a periodic vector population. Bulletin of Mathematical Biology 69: 1067–1091. 17265121 10.1007/s11538-006-9166-9

[2] Bacaër N, Guernaoui S (2006) The epidemic threshold of vector-borne diseases with seasonality. Journal of mathematical biology 53: 421–436. 16823580 10.1007/s00285-006-0015-0

[3] Bacaër N, Ouifki R (2007) Growth rate and basic reproduction number for population models with a simple periodic factor. Mathematical Biosciences 210: 647–658. 17822724 10.1016/j.mbs.2007.07.005

[4] Smith DL, Hay SI, Noor AM, Snow RW (2009) Predicting changing malaria risk after expanded insecticide-treated net coverage in Africa. Trends in Parasitology 25: 511–516. 10.1016/j.pt.2009.08.002 19744887 PMC2768685

[5] Killeen GF, Chitnis N, Moore SJ, Okumu FO (2011) Target product profile choices for intra-domiciliary malaria vector control pesticide products: repel or kill? Malaria Journal 10 10.1186/1475-2875-10-207 21798023 PMC3199905

[6] Griffin JT, Ferguson NM, Ghani AC (2014) Estimates of the changing age-burden of Plasmodium falciparum malaria disease in sub-Saharan Africa. Nat Commun 5 10.1038/ncomms4136 24518518 PMC3923296

[7] Griffin JT, Hollingsworth TD, Okell LC, Churcher TS, White M, et al. (2010) Reducing Plasmodium falciparum malaria transmission in Africa: a model-based evaluation of intervention strategies. PLoS Med 7: e1000324 10.1371/journal.pmed.1000324 20711482 PMC2919425

[8] Gu W, Killeen GF, Mbogo CM, Regens JL, Githure JI, et al. (2003) An individual-based model of Plasmodium falciparum malaria transmission on the coast of Kenya. Transactions of The Royal Society of Tropical Medicine and Hygiene 97: 43–50. 12886804 10.1016/s0035-9203(03)90018-6

[9] Okell LC, Griffin JT, Kleinschmidt I, Hollingsworth TD, Churcher TS, et al. (2011) The Potential Contribution of Mass Treatment to the Control of Plasmodium falciparum Malaria. Plos One 6 10.1371/journal.pone.0020179 21629651 PMC3101232

[10] Crowell V, Briet O, Hardy D, Chitnis N, Maire N, et al. (2013) Modelling the cost-effectiveness of mass screening and treatment for reducing Plasmodium falciparum malaria burden. Malaria Journal 12: 4 10.1186/1475-2875-12-4 23286228 PMC3544609

[11] Smith DL, McKenzie FE, Snow RW, Hay SI (2007) Revisiting the basic reproductive number for malaria and its implications for malaria control. PLoS Biology 5: 531–542. 10.1371/journal.pbio.0050042 17311470 PMC1802755

[12] Gosling RD, Okell L, Mosha J, Chandramohan D (2011) The role of antimalarial treatment in the elimination of malaria. Clinical Microbiology and Infection 17: 1617–1623. 10.1111/j.1469-0691.2011.03660.x 21951597

[13] Diekmann O, Heesterbeek H, Britton T (2012) Mathematical tools for understanding infectious disease dynamics: Princeton University Press.

[14] Dye C, Hasibeder G (1986) Population dynamics of mosquito-borne disease: effects of flies which bite some people more frequently than others. Transactions of the Royal Society of Tropical Medicine and Hygiene 80: 69–77. 3727001 10.1016/0035-9203(86)90199-9

[15] Bacaër N, Ait Dads E (2011) Genealogy with seasonality, the basic reproduction number, and the influenza pandemic. Journal of Mathematical Biology 62: 741–762. 10.1007/s00285-010-0354-8 20607242

[16] Householder AS (2013) The Theory of Matrices in Numerical Analysis: Dover Publications.

[17] Johnston GL, Smith DL, Fidock DA (2013) Malaria’s Missing Number: Calculating the Human Component of R0 by a Within-Host Mechanistic Model of Plasmodium falciparum Infection and Transmission. PLoS Comput Biol 9: e1003025 10.1371/journal.pcbi.1003025 23637586 PMC3630126

[18] White NJ (2005) Intermittent Presumptive Treatment for Malaria. PLoS Med 2: e3 10.1371/journal.pmed.0020003 15696210 PMC545196

[19] White MT, Griffin JT, Churcher TS, Ferguson NM, Basanez M-G, et al. (2011) Modelling the impact of vector control interventions on Anopheles gambiae population dynamics. Parasites & Vectors 4 10.1186/1756-3305-4-153 21798055 PMC3158753

[20] Le Menach A, Takala S, McKenzie FE, Perisse A, Harris A, et al. (2007) An elaborated feeding cycle model for reductions in vectorial capacity of night-biting mosquitoes by insecticide-treated nets. Malaria Journal 6: 10 10.1186/1475-2875-6-10 17254339 PMC1794417

[21] Bejon P, White MT, Olotu A, Bojang K, Lusingu JPA, et al. (2013) Efficacy of RTS,S malaria vaccines: individual-participant pooled analysis of phase 2 data. The Lancet Infectious Diseases 13: 319–327. 10.1016/S1473-3099(13)70005-7 23454164 PMC3771416

[22] Molineaux L, Gramiccia G (1980) The Garki project: research on the epidemiology and control of malaria in the Sudan savanna of West Africa: WHO. 311p p.

[23] Shiff C, Minjas J, Hall T, Hunt R, Lyimo S, et al. (1995) Malaria infection potential of anopheline mosquitoes sampled by light trapping indoors in coastal Tanzanian villages. Medical and veterinary entomology 9: 256–262. 7548942 10.1111/j.1365-2915.1995.tb00131.x

[24] Kasasa S, Asoala V, Gosoniu L, Anto F, Adjuik M, et al. (2013) Spatio-temporal malaria transmission patterns in Navrongo demographic surveillance site, northern Ghana. Malaria Journal 12: 63 10.1186/1475-2875-12-63 23405912 PMC3618087

[25] Gadiaga L, Machault V, Pages F, Gaye A, Jarjaval F, et al. (2011) Conditions of malaria transmission in Dakar from 2007 to 2010. Malaria Journal 10: 312 10.1186/1475-2875-10-312 22018223 PMC3216462

[26] R Core Team (2013) R: A language and environment for statistical computing. Vienna, Austria: R Foundation for Statistical Computing.

